# *In Vivo* Antiplasmodial Potentials of the Combinations of Four Nigerian Antimalarial Plants

**DOI:** 10.3390/molecules190913136

**Published:** 2014-08-26

**Authors:** Adeleke Clement Adebajo, Samuel Akintunde Odediran, Fatimah Abosede Aliyu, Paul Alozie Nwafor, Ndifreke Thomas Nwoko, Usenobong Samuel Umana

**Affiliations:** 1 Department of Pharmacognosy, Faculty of Pharmacy, Obafemi Awolowo University, Ile-Ife 220282, Osun State, Nigeria; 2 Department of Pharmacognosy and Herbal Medicine, Faculty of Pharmacy, University of Uyo, Uyo 520003, Akwa Ibom State, Nigeria; 3 Department of Pharmacology and Toxicology, Faculty of Pharmacy, University of Uyo, Uyo 520003, Akwa Ibom State, Nigeria

**Keywords:** Nigerian ethnomedicinal plants, *in vivo* antimalarial models, decoctions, herbal combinations, synergism

## Abstract

Various combinations of *Nauclea latifolia* root, *Artocarpus altilis* stem bark, *Murraya koenigii* leaf and *Enantia chlorantha* stem bark used in African ethnomedicine as decoctions for malaria and fevers, and combinations with standard drugs, were investigated for antiplasmodial activities using *Plasmodium berghei berghei-*infected mice. The respective prophylactic and curative ED_50_ values of 189.4 and 174.5 mg/kg for *N. latifolia* and chemosuppressive ED_50_ value of 227.2 mg/kg for *A. altilis* showed that they were the best antimalarial herbal drugs. A 1.6-fold increase of the survival time given by the negative control was elicited by *M. koenigii*, thereby confirming its curative activity. Pyrimethamine with an ED_50_ of 0.5 ± 0.1 mg/kg for the prophylactic, and chloroquine with ED_50_ = 2.2 ± 0.1 and 2.2 ± 0.0 mg/kg for the chemosuppressive and curative tests, respectively, were significantly (*p* < 0.05) more active. Co-administrations of *N. latifolia* with the standard drugs significantly reduced their prophylactic, chemosuppressive and curative actions, possibly increasing the parasites’ resistance. Binary combinations of *N. latifolia* or *M. koenigii* with any of the other plants significantly increased the prophylactic and suppressive activities of their individual plants, respectively. Also, *E. chlorantha* with *A. altilis* or *N. latifolia* enhanced their respective prophylactic or curative activities, making these combinations most beneficial against malaria infections. Combinations of three and four extracts gave varied activities. Hence, the results justified the combinations of ethnomedicinal plants in antimalarial herbal remedies and showed the importance of the three *in vivo* models in establishing antimalarial activity.

## 1. Introduction

Malaria is a major tropical disease with high morbidity and mortality, especially in the sub-Saharan Africa, Asia and Latin America continents [1]. About 3.4 billion people worldwide are exposed annually, with 1.2 billion at high risk and some 207 million developed symptomatic malaria in 2012. The number of deaths, which occurred mainly among children and pregnant women, fell from 1.82 million in 2004 to 1.24 million in 2010 [2]. The sufferers are found in both urban and rural undeveloped communities. Although malaria is not currently listed among the WHO's neglected diseases, the fact that these rural or “neglected” populations lack access to orthodox medical practices due to non-availability or their high poverty status, resulting in high mortality among them, makes malaria a major health problem. Therefore, within the tropical and poor countries of the world housing such “neglected populations”, the nomenclature of “neglected disease” for malaria would be appropriate. Hence, most of these “rural/neglected populations” depend largely on the traditional medical practitioners for their cure, using herbal decoctions or potions [3,4,5,6]. These decoctions, being combinational, are believed to combat the increasing resistance of the malarial *Plasmodium* parasites to chloroquine and other orthodox drugs employed in its treatment. Similarly, in orthodox medicine, Artemisinin Combination Therapy (ACT) is the treatment of choice for malaria [4,5,6]. Stem and root of *Nauclea latifolia* Smith (Rubiaceae) and stem barks of *Enantia chlorantha* Oliv. (Annonaceae) and *Artocarpus altilis* (Parkinson) Fosberg (Moraceae) are employed folklorically to treat malaria and other febrile conditions in the humid tropics [7], including Nigeria and Cameroon [8]. A leaf infusion of *Murraya koenigii* (Linn.) Sprengor (Rutaceae) is drunk in northern Nigeria to treat fevers [9].

Five prenylflavonoids and triterpenoids were previously isolated from the root and seed oil of *A. altilis*, and the moderate *in vitro* antiplasmodial activities of these flavonoids identified them as the active constituents [10]. Antimalarial activities of *A. altilis* root and stem have also been reported [10]. *In vitro* antiplasmodial activity of *N. latifolia* stem and root have been demonstrated, while indole alkaloids from its bark and wood, strictosamine and loganin from stem and root and two saponins from the bark were isolated [7,11,12]. Using *Plasmodium yoelii-*infected mice, aqueous extract of *E. chlorantha* had both suppressive (ED_50_ = 6.9 g/kg) and prophylactic antiplasmodial actions, while the ethanolic extract, with an ED_50_ of 0.34 g/kg, had only curative action [13]. This activity was suggested to be due to its contents of berberine, saponins and tannins [14]. The protoberberine alkaloids berberine, palmatine and jatrorrhizine were identified as potential active principles because they demonstrated *in vitro* activity against *P. falciparum* that was comparable (*p* > 0.05) to quinine, although they lacked any *in vivo* activity against *P. berghei* [15]. Numerous monomeric and dimeric carbazole alkaloids have been reported for *M. koenigii* leaf [9], which has been reported to give a dose-dependent antimalarial activity against *P. berghei* chloroquine-resistant NK65 strain [16]. Bioassay-guided chromatographic fractionation of its leaf ethyl acetate extract, using the *in vitro* and chemosuppresive *in vivo* antimalarial assay methods, yielded myristic acid and β-caryophyllene as its antimalarial constituents [16].

The World Health Organization [17] recommendation of ACT as a policy of malaria treatment, may be an impetus for the continued investigation of combinations of herbs with reported ethnomedicinal antimalarial usage as a promising approach for discovery of candidate drugs [4,18]. Such drugs must have reports of both *in vivo* and *in vitro* anti-plasmodial activities [19]. Hence, the potentials of *A. altilis*, *N. latifolia* and *M. koenigii*, as possible components of herbal decoctions, were evaluated in this study, using their aqueous-ethanolic extracts, *Plasmodium berghei berghei*-infected mice and the three *in vivo* prophylactic, chemosuppresive and curative models, with the aims of corroborating their *in vitro* antimalarial activities and providing scientific evidence supporting their ethnomedicinal usage. Also, the curative activity of *E. chlorantha* was re-evaluated.

## 2. Results and Discussion

Antiplasmodial activities of the four plants were evaluated against *P. berghei berghei*, using the *in vivo* prophylactic, chemosuppressive and curative test models, in order to validate their ethnomedicinal claims and determine the potentials of their various combinations, and also of combinations with standard drugs. Considering the ED_50_ and ED_90_ values of the extracts, *N. latifolia* had the best prophylactic and curative activities, while *A. altilis* was the best chemosuppressive drug. In all the models, the activities of the standard drugs, pyrimethamine (**PYR**) or chloroquine (**CQ**) were significantly better than those of the extracts (Table 1). 

**Table 1:** *In vivo* antimalarial activities of the four Nigerian ethnomedicinal plants.

Extract/Drug	Antimalarial Activity (mg/kg) per Model Type					
	ED_50_			ED_90_		
	PRO	SUP	CUR	PRO	SUP	CUR
*Nauclea latifolia* (Root)	189.4 ± 2.9 ^b^	279.3 ± 2.9 ^c^	174.5 ± 1.3 ^b^	356.0 ± 5.1 ^b^	497.2 ± 4.5 ^d^	325.2 ± 1.7 ^b^
*Arthocarpus altilis* (Stem bark)	214.2 ± 1.0 ^d^	227.2 ± 0.3 ^b^	310.2 ± 2.1 ^d^	454.3 ± 1.8 ^d^	373.0 ± 0.5 ^b^	465.6 ± 3.8 ^e^
*Murraya koenigii* (Leaf)	195.6 ± 0.9 ^c^	287.1 ± 0.7 ^d^	252.4 ± 1.6 ^c^	374.4 ± 1.5 ^c^	397.6 ± 1.2 ^c^	450.1 ± 2.9 ^d^
*Enantia chlorantha* (Stem bark)	NT	NT	317.9 ± 2.8 ^e^	NT	NT	432.0 ± 6.8 ^c^
PC (positive controls)	0.5 ± 0.1 ^a^	2.2 ± 0.1 ^a^	2.2 ± 0.0 ^a^	0.9 ± 0.2 ^a^	4.3 ± 0.2 ^a^	4.1 ± 0.0 ^a^

Data show the mean ± SEM, *n* = 5. **ED**_50_, **ED**_90_: Doses that produced 50% and 90% activities; **PRO**, **SUP**, **CUR**: Prophylactic, Chemosuppressive, Curative models, respectively; PC (positive controls): Pyrimethamine (**PYR**) for prophylactic, and Chloroquine (**CQ**) for chemosuppressive and curative models. a, b, c, d, e: Values with different superscripts within columns are significantly different (*p* < 0.05, one-way analysis of variance followed by the Student–Newman–Keuls’ test).

Hence, *N. latifolia* and *M. koenigii* would be effective at all stages of malaria treatment. Antimalarial drugs with good prophylactic activity, when absorbed into the blood stream, should prevent the invasion of the liver by the sporozoites, while those with good chemosuppressive activity should suppress the development of the merozoites to schizonts in the liver and their release into the blood as trophozoites. Those with good curative activity should destroy the trophozoites in the blood and/or prevent formation of gametocytes, thereby preventing re-infection of the mosquitoes and man [20].

The rural dwellers of the African, Asian and Latin America continents consult traditional practitioners as their first choice of medical care and only visit the hospital or qualified medical personnel when there is no relief. Even on the hospital beds, herbal drugs are secretly taken together with their prescribed drugs. Hence, the investigation of the effects of co-administering herbal and orthodox drugs was imperative, using the extracts’ median doses (ED_50_), and **CQ** or **PYR** at 10.0 or 1.2 mg/kg, respectively, in order to ascertain the possible effects of such practice on the activities of standard drugs employed for the treatment of malaria in the hospitals.

Generally, the co-administrations of the individual extracts with standard drugs gave activities that were similar (*p* > 0.05) or lower than those of **CQ** or **PYR**. The co-administration of **CQ** with *N. latifolia* or *A. altilis* or *E. chlorantha* significantly reduced the curative activity of **CQ** while only *N. latifolia* inhibited the prophylactic activity of **PYR** and suppressive activity of **CQ** (Table 2), indicating that combining these herbal drugs with **CQ** or **PYR** would increase the development of the parasites’ resistance. Only *M. koenigii* did not significantly affect the prophylactic, chemosuppressive and curative effects of **CQ** (Table 2).

**Table 2:** *In vivo* antimalarial activities of the four Nigerian ethnomedicinal plants combined with standard drugs.

Extract/Drug *	Percentage Reduction in Parasitaemia per Model Type		
	Prophylactic	Chemosuppressive	Curative
NC (negative control)	0.0 ± 2.4 ^a^	0.0 ± 1.3 ^a^	0.0 ± 1.6 ^a^
*Nauclea latifolia* (root)	55.6 ± 1.0 ^c^	34.4 ± 1.5 ^b^	51.1 ± 0.8 ^b^
*Nauclea latifolia* (root) + PC	80.5 ± 0.6 ^d^	83.3 ± 0.5 ^d^	76.6 ± 0.9 ^d^
*Arthocarpus altilis* (stem bark)	48.7 ± 0.7 ^b^	55.5 ± 0.2 ^c^	50.4 ± 0.6 ^b^
*Arthocarpus altilis* (stem bark) + PC	95.7 ± 0.4 ^e^	94.4 ± 0.3 ^e^	82.3 ± 1.0 ^e^
*Murraya koenigii* (leaf)	56.2 ± 0.0 ^c^	48.5 ± 0.2 ^b^	58.1 ± 0.0 ^c^
*Murraya koenigii* (leaf) + PC	94.1 ± 0.1 ^e^	98.2 ± 0.3 ^f^	100.0 ± 0.0 ^g^
*Enantia chlorantha* (stem bark)	53.5 ± 0.3 ^c^	56.4 ± 0.2 ^d^	49.7 ± 0.4 ^b^
*Enantia chlorantha* (stem bark) + PC	95.2 ± 0.3 ^e^	96.1 ± 0.3 ^e,f^	81.2 ± 0.4 ^e^
PC (positive controls)	96.4 ± 0.1 ^e^	97.0 ± 0.1 ^e,f^	99.3 ± 0.0 ^f^

Data show the mean ± SEM, *n* = 5. *: Doses of the extracts given were those that gave 50% activity; NC (negative control): Tween 80 in normal saline; PC (positive controls): Pyrimethamine (1.2 mg/kg) for prophylactic, and Chloroquine (10 mg/kg) for chemosuppressive and curative models. a, b, c, d, e, f, g: Values with different superscripts within columns are significantly different (*p* < 0.05, one-way analysis of variance followed by the Student–Newman–Keuls’ test).

The co-administration of standard drugs with *N. latifolia* in the prophylactic and suppressive models, with *A. altilis* in the prophylactic model, and with *E. chlorantha* in the prophylactic and curative models significantly extended the survival times of the mice compared to those given the individual extracts. The percentages of these elongations over those given by their respective negative controls were also comparable to those elicited by the standard drugs (Table 3). In the curative model, only co-administration of *E. chlorantha* with **CQ** gave 168.2% ± 50.9%, that was more than double [21] the 67.3% ± 20.0% prolongation of the lives of the mice given by its extract alone, although this was just 1.6 times the 100.0% ± 8.2% elicited by normal saline (Table 3). Also, the percentages of the survival times given by *N. latifolia* and *M. koenigii* as well as the combinations of **CQ** with either *N. latifolia* or *E. chlorantha* or *M. koenigii* were comparable to those elicited by the standard drugs (Table 3). There was a correlation only between the curative activity and elongation of the survival times in the mice co-administered with **CQ** + *M. koenigii* while the discrepancies observed in the combinations of **CQ** with other extracts (Table 2 and Table 3) may indicate that other properties of the extracts, such as immune enhancement, may be responsible for their increased survival times [4].

**Table 3:** Survival times of mice treated with the four Nigerian ethnomedicinal plants and combinations of the plants with standard drugs.

Extract/Drug *	Survival time as Percentage of Negative Control per Model Type		
	Prophylactic	Chemosuppressive	Curative
NC (negative control)	100.0 ± 6.3 ^a^	100.0 ± 15.8 ^a^	100.0 ± 8.2 ^a,b^
*Nauclea latifolia* (root)	312.5 ± 66.1 ^b,c,d^	115.8 ± 19.3 ^a^	195.5 ± 4.6 ^b^
*Nauclea latifolia* (root) + PC	437.5 ± 0.0 ^d^	184.2 ± 0.0 ^a^	181.1 ± 27.2 ^b^
*Arthocarpus altilis* (stem bark)	287.5 ± 54.7 ^b,c,d^	100.0 ± 29.8 ^a^	69.1 ± 30.0 ^a^
*Arthocarpus altilis* (stem bark) + PC	437.5 ± 0.0 ^d^	143.9 ± 37.7 ^a^	94.6 ± 5.5 ^a,b^
*Murraya koenigii* (leaf)	212.5 ± 60.9 ^a,b^	119.3 ± 34.2 ^a^	160.0 ± 18.2 ^a,b^
*Murraya koenigii* (leaf) + PC	290.6 ± 54.7 ^b,c,d^	115.8 ± 10.5 ^a^	170.0 ± 0.9 ^a,b^
*Enantia chlorantha* (stem bark)	240.6 ± 48.4 ^a,b,c^	98.3 ± 21.9 ^a^	67.3 ± 20.0 ^a^
*Enantia chlorantha* (stem bark) + PC	396.9 ± 40.6 ^c,d^	157.9 ± 53.5 ^a^	168.2 ± 50.9 ^a,b^
PC (positive controls)	437.5 ± 0.0 ^d^	184.2 ± 0.0 ^a^	163.6 ± 27.3 ^a,b^

**Keys**: Data show the mean ± SEM, *n* = 5. *: Doses of the extracts given were those that gave 50% activity; NC (negative control): Tween 80 in normal saline; PC (positive controls): Pyrimethamine (1.2 mg/kg) for prophylactic, and Chloroquine (10 mg/kg) for chemosuppressive and curative models. a, b, c, d: Values with different superscripts within columns are significantly different (*p* < 0.05, one-way analysis of variance followed by the Student–Newman–Keuls’ test).

Three decoctions consisting of barks of *E. chlorantha*, *Alstonia boonei* and *Diospyros mespiliformis*; *E. chlorantha* bark with rhizomes of *Curcuma longa*; and barks of *E. chlorantha* and *A. boonei* are usually taken as antimalarial recipes in Oke-Igbo, Nigeria [3], implying that *E. chlorantha*, like some other plants, has the potential of being combined in an antimalarial remedy. Therefore, various combinations of the extracts of two, three or four of the plants used in this study were given to the mice. The dosage of each extract in each combination was that equal to its individual ED_50_ value in that particular model.

Co-administration of *N. latifolia* (**NL**) with either *A. altilis* (**AA**) or *M. koenigii* (**MK**) or *E. chlorantha* (**EC**) significantly improved their prophylactic activity (Table 4) over the individual extracts (Table 2) while **NL** combined with **EC** significantly gave higher curative activity (Table 2 and Table 4). Similarly, **MK** + **NL**, **MK** + **EC** and **MK** + **AA** gave significantly enhanced suppressive activities over their individual plants. These increased effects observed with the decoctions over the activities of the individual herbal extracts may suggest synergism in their activities. Similar synergistic effects have recently been reported for some decoctions of Nigerian herbs [4]. On the other hand, **AA** + **NL**, **AA** + **MK** or **AA** + **EC** combinations elicited significantly reduced curative activities than their individual extracts, while combining **MK** with **EC** resulted in reduced prophylactic and curative activities (Table 2 and Table 4). Hence, caution must be exercised in the choice of ethnomedicinal plants to be combined in traditional decoctions for the treatment of malaria, as not all combinations have increased benefits to the users as antimalarial drugs [4].

The tripartite combination of **NL** + **AA** + **EC** gave increased prophylactic and suppressive activities over those of the **NL** + **AA**, **NL** + **EC** and **AA** + **EC** dual combinations, confirming synergism in the antimalarial actions of the plants. Also, addition of either **NL** to **MK**+**EC** or **EC** to **NL** + **MK** to obtain the **NL** + **MK** + **EC** combination significantly increased their curative activities to 80%, which was the same value given by **NL** + **EC** (Table 4). Therefore, in this case, the addition of **MK** to the latter combination had no curative antimalarial beneficial effect. Furthermore, the combination of the four plants (**NL** + **AA** + **MK** + **EC**) gave significantly lowered suppressive activity compared to the individual plants, their **2-** and **3-**combinations

**Table 4:** Antimalarial activities of multiple combinations of the four Nigerian medicinal plants.

Extract/Drug *	Percentage Reduction in Parasitaemia per Model Type		
	Prophylactic	Chemosuppressive	Curative
NC (negative control)	0.0 ± 2.4 ^a^	0.0 ± 1.3 ^b^	0.0 ± 1.6 ^b^
NL+AA	75.0 ± 1.4 ^g^	44.5 ± 6.9 ^d^	- ^a^
NL+MK	58.4 ± 1.1 ^e^	78.9 ± 0.2 ^g^	56.9 ± 0.9 ^f^
NL+EC	68.9 ± 2.9 ^f^	42.4 ± 3.1 ^d^	79.5 ± 3.7 ^g^
AA+MK	49.5 ± 0.8 ^d^	64.2 ± 1.2 ^f^	- ^b^
AA+EC	56.6 ± 4.0 ^e^	56.4 ± 4.3 ^e^	38.0 ± 1.2 ^d,e^
MK+EC	16.7 ± 1.1 ^c^	76.9 ± 0.2 ^g^	29.8 ± 4.8 ^c,d^
NL+AA+MK	7.6 ± 2.9 ^b^	28.5 ± 1.4 ^c^	24.7 ± 1.0 ^c^
NL+AA+EC	87.4 ± 1.4 ^h^	65.7 ± 2.5 ^f^	- ^b^
NL+MK+EC	47.5 ± 1.4 ^d^	23.5 ± 0.4 ^c^	79.8 ± 0.4 ^g^
AA+MK+EC	50.3 ± 1.0 ^d^	73.1 ± 1.4 ^f,g^	20.2 ± 1.0 ^c^
NL+AA+MK+EC	60.2 ± 0.5 ^e^	- ^a^	52.7 ± 2.3 ^f^
PC (positive controls)	96.4 ± 0.1 ^i^	97.0 ± 0.1 ^h^	99.3 ± 0.0 ^h^

Data show the mean ± SEM, *n* = 5. *: Doses of the extract given were those that gave 50% activity; -: No reduction in parasitaemia; NC (negative control): Tween 80 in normal saline; NL: *Nauclea latifolia* (root); AA: *Arthocarpus altilis* (stem bark); MK: *Murraya koenigi* (leaf); EC: *Enantia chlorantha* (stem bark); PC (positive controls): Pyrimethamine (1.2 mg/kg) for prophylactic, and Chloroquine (10 mg/kg) for chemosuppressive and curative models. a, b, c, d, e, f, g, h, i: Values with different superscripts within columns are significantly different (*p* < 0.05, one-way analysis of variance followed by the Student–Newman–Keuls’ test).

The survival times elicited by these numerous combinations in the treated mice were also significantly lower than those of animals treated with the standard drugs (Table 5), confirming their lower antimalarial activities. Hence, the totality of these results showed that the plant components of a decoction are crucial to its ethnomedicinal usefulness in preventing, suppressing or curing malaria infection. Similar observations of synergism and the effects of the ratio of the plant components on the prophylactic, chemosuppressive and curative activities of MAMA decoction, an antimalarial herbal remedy, commonly prepared and used in Nigeria from 1:1:1:1 ratio of *Mangifera indica*, *Alstonia boonei*, *Morinda lucida*, and *Azadirachta indica* leaves and its four combination variants, MAMA-1, -2, -3 and -4, consisting of 1:2:2:2; 2:1:2:2; 2:2:2:1 and 1:1:2:2 ratios of the above plants, respectively were recently made [4]. Finally, the combinations of the extracts of the two, three and four plants gave prophylactic, suppressive and curative activities that were significantly lower than those of the standard drugs. Despite their lowered potencies, these results justified the traditional usage of these plants as antimalarial remedies. Also, some cases of synergistic or inhibitory effects between the plant-drugs were demonstrated (Table 4). 

**Table 5:** Survival times of mice given multiple combinations of the four Nigerian medicinal plants.

Extract/Drug *	Survival Time as Percentage of Negative Control per Model Type		
	Prophylactic	Chemosuppressive	Curative
NC (negative control)	100.0 ± 6.3 ^a,b^	100.0 ± 15.8 ^a,b^	100.0 ± 8.2 ^a,b^
NL+AA	128.1 ± 34.4 ^a,b^	101.8 ± 29.8 ^a,b^	86.4 ± 8.2 ^a,b^
NL+MK	267.2 ± 10.9 ^b^	121.1 ± 10.5 ^a,b^	112.7 ± 7.3 ^a,b^
NL+EC	118.8 ± 34.4 ^a,b^	56.1 ± 14.0 ^a^	89.1 ± 4.6 ^a,b^
AA+MK	256.3 ± 28.1 ^b^	128.1 ± 36.0 ^a,b^	56.4 ± 8.2 ^a^
AA+EC	78.1 ± 25.0 ^a^	131.6 ± 30.7 ^a,b^	83.6 ± 6.4 ^a,b^
ML+EC	231.3 ± 67.2 ^a,b^	101.8 ± 19.3 ^a,b^	74.6 ± 15.5 ^a,b^
NL+AA+MK	209.4 ± 21.9 ^a,b^	122.8 ± 27.2 ^a,b^	103.6 ± 4.6 ^a,b^
NL+AA+EC	181.3 ± 78.1 ^a,b^	73.7 ± 22.8 ^a,b^	83.6 ± 6.4 ^a,b^
NL+MK+EC	159.4 ± 25.0 ^a,b^	103.5 ± 20.2 ^a,b^	92.7 ± 14.6 ^a,b^
AA+MK+EC	268.9 ± 12.5 ^b^	84.2 ± 35.1 ^a,b^	112.7 ± 8.2 ^a,b^
NL+AA+MK+EC	221.9 ± 25.0 ^a,b^	87.7 ± 29.8 ^a,b^	129.1 ± 14.6 ^b^
PC (positive controls)	437.5 ± 0.0 ^c^	184.2 ± 0.0 ^b^	163.6 ± 27.3 ^c^

Data show the mean ± SEM, *n* = 5. *: Doses of the extract given were those that gave 50% activity; NC (negative control): Tween 80 in normal saline; NL: *Nauclea latifolia* (root); AA: *Arthocarpus altilis* (stem bark); MK: *Murraya koenigi* (leaf); EC: *Enantia chlorantha* (stem bark); PC (positive controls): Pyrimethamine (1.2 mg/kg) for prophylactic, and Chloroquine (10 mg/kg) for chemosuppressive and curative models^.^ a, b, c: Values with different superscripts within columns are significantly different (*p* < 0.05, one-way analysis of variance followed by the Student–Newman–Keuls’ test).

## 3. Experimental Section

### 3.1. Plant Collection and Extraction

Fresh roots of *N. latifolia* (**NL**) were collected in November, 2010 from Abiakana, Akwa Ibom State, Nigeria from trees already authenticated by Dr. (Mrs) Margaret Bassey (Department of Botany and Ecological Studies, University of Uyo) while the stem bark of *A. altilis* (**AA**) and *M. koenigii* (**MK**) leaves were also collected in March 2011 near the gate and in the staff quarters of Obafemi Awolowo University, Ile-Ife, respectively from trees already authenticated by H. C. Illoh, (Department of Botany, Obafemi Awolowo University, Ile-Ife), respectively. Stem bark of *E. chlorantha* (**EC**) was purchased in November 2010 from the Itakogun market in Ile-Ife. Their respective voucher specimens UUH1666/IKA, IFE16545, FHI 105244 and FPI-1876 were deposited in the herbaria of these universities and that of Forestry Research Institute of Nigeria, Ibadan. An amount of 500 g of **NL** and 100 g each of **AA**, **EC** and **MK** were separately extracted with 70% ethanol (5 L) at room temperature in a percolator for 72 hours, with occasional agitation. The extracts were filtered and concentrated *in vacuo* to give their respective dried ethanolic extracts coded **NL** (178.4 g), **AA** (14.5 g), **EC** (15.05 g), and **MK** (102.0 g) and yields of 13.1, 14.5, 15.1 and 10.1% w/w, respectively and stored in the refrigerator (4 °C) until needed.

### 3.2. Animals and Parasites

Healthy Wistar albino mice (18–22 g) of either sex, maintained in separate cages at 22 °C under natural 12 h daylight/night conditions in the animal houses of University of Uyo and Obafemi Awolowo University and acclimatized for at least 5 days preceeding the experiments, were fed on standard pellet diet (Bendel Feeds, Benin , Nigeria) and water was given *ad libitum.* The “principle of laboratory animal care” (NIH publication No. 85-23, 1985) guidelines and procedures [22] were followed for the experiments. Rodent parasites used were *P. berghei berghei* NK65 obtained from the Institute of Medical Research and Training, University College Hospital, Ibadan, Nigeria.

### 3.3. In Vivo Antiplasmodial Activities of the Individual Plants

Suspensions of the extracts and pyrimethamine were prepared by triturating weighed samples in tragacanth and diluting with distilled water to give a 1% final concentration of tragacanth, while chloroquine phosphate was dissolved in distilled water. A total of three hundred mice were used for the ten experiments (Table 1,Table 2 and Table 3). The prophylactic, chemosuppressive and curative antiplasmodial activities of the aqueous-ethanolic extracts of *N. latifolia*, *A. altilis* and *M. koenigii* as well as the curative activity of *E. chlorantha* were assessed by the methods described by Peters, and Ryley and Peters [23,24]. Extracts of *N. latifolia*, or *A. altilis*, or *M. koenigii* or *E. chlorantha* (100, 200, 400 mg/kg), or standard drugs of **CQ** (10 mg/kg) or **PYR** (1.2 mg/kg), or the vehicle (negative control), were administered (*p.o.*), using oral cannula, to groups of five mice each, daily for 3 days before infection in the prophylactic model. Extracts/drugs were also given *p.o.* two hours after infection and thereafter daily for 3 days in the chemosuppressive model while in curative model, administration was done daily for 5 days, starting from the third day after infection. The blood films of the mice were respectively taken at 3 and 4 days and daily after infection for these 3 models. Percentage parasitaemia, determined by counting 10 fields of the blood smear in a view under the microscope, was used to calculate percentages reduction in parasitaemia, chemosupression and clearance for each dose, using the formula:

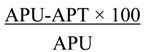

where APU is average percentage parasitaemia in the untreated mice and APT is average percentage parasitaemia in the treated mice [23]. Microsoft Excel 2007 was used to forecast the ED_50_ and ED_90_ values of the extracts and standard drugs, as a measure of their activities.

### 3.4. In Vivo Antiplasmodial Activities of the Various Combinations of the Plants with Standard Drugs

The doses that gave 50% of the respective activities (ED_50_) of the extracts were administered *p.o.* in quick succession with the standard drugs to the groups of mice and their prophylactic, chemosuppressive and curative antiplasmodial activities were determined, as given above. Also, the prophylactic, chemosuppressive and curative antiplasmodial activities of combinations of the aqueous-ethanolic extracts of the two plants as: **NL** + **AA**, **NL** + **EC**, **NL** + **MK**, **AA** + **EC**, **AA** + **MK**, **MK** + **EC**; three plants as: **NL** + **AA** + **EC**, **NL** + **AA** + **MK**, **NL** + **MK** + **EC**, **AA** + **MK** + **EC**, and of four plants as: **NL** + **AA** + **MK** + **EC** were assessed by administering each in quick succession to the groups of mice, the median effective doses (ED_50_) in the three models. A total of one hundred and ninety-five mice were used for the thirteen experiments (Table 4 and Table 5).

### 3.5. Survival Times

The mean survival time of the mice in each treatment group was also determined arithmetically by finding the average survival time (days) of the mice post inoculation in each group over a period of 28 days.

### 3.6. Statistical Analysis

The percentage parasitaemia, percentage clearance, percentage chemosupression, percentage reduction in parasitaemia, ED_50_ and ED_90_ values and survival times were expressed as mean ± SEM. The significance of comparative difference was determined between the extracts/standard drugs/combinations and the negative control using One-Way Analysis of Variance (ANOVA), followed by Student–Neumann–Keuls *post-hoc* test. Values of *p* < 0.05 were considered statistically significant.

## 4. Conclusions

In conclusion, although the activities of these four plants appear low in comparison with the standard drugs, they show significant antimalarial effects in the various models investigated in this study, which can be interpreted as a justification for their antimalarial ethnomedicinal claims. Studies on the chemical constituents responsible for these effects will be interesting and might lead to new chemical entities with antimalarial potential. The present results obtained by three complementary *in vivo* models furthermore show the importance of the choice of plant components used to prepare herbal remedies to prevent, suppress and cure malaria infestation. 
